# The Relationship between Erectile Dysfunction, Self-Esteem, and Depression in Post-Myocardial Infarction Patients

**DOI:** 10.3390/jcm13206134

**Published:** 2024-10-15

**Authors:** Oskar Wróblewski, Edyta Skwirczyńska, Kaja Michalczyk, Samir Zaeir, Labib Zair, Klara Kraszewska, Julia Kiryk, Alicja Bobik, Anna Mikołajczyk-Kocięcka, Anita Chudecka-Głaz

**Affiliations:** 1 Department of Gynecological Surgery and Gynecological Oncology of Adults and Adolescents, Pomeranian Medical University, 70-204 Szczecin, Poland; oskraw@gmail.com (O.W.); socjoterapia@onet.pl (E.S.); anitagl@poczta.onet.pl (A.C.-G.); 2 Collegium Medicum, University of Zielona Góra, 65-417 Zielona Gora, Poland; 3 General and Transplant Surgery Ward with Sub-Departments of Pomeranian Regional Hospital in Szczecin, 71-455 Arkonska, Poland; samizeair@gmail.com; 4 Department of General Surgery and Transplantation, Pomeranian Medical University, 70-204 Szczecin, Poland; labibzair76@gmail.com; 5 Faculty of Pharmacy, Medical Biotechnology and Laboratory Medicine, Pomeranian Medical University, 70-204 Szczecin, Poland; k.kraszewska293@wp.pl (K.K.); julka.kiryk@wp.pl (J.K.); alxebx@gmail.com (A.B.); 6 Department of Medical Rehabilitation and Clinical Rehabilitation, Pomeranian Medical University in Szczecin, Żołnierska 54, 71-210 Szczecin, Poland; mikolajczyk.ania1@wp.pl

**Keywords:** erectile dysfunction, myocardial infarction, cardiac rehabilitation, depression, self-esteem

## Abstract

**Background:** The interplay between the physical and mental health of patients recovering from myocardial infarction (MI) is crucial. Erectile dysfunction (ED) is a common sexual issue, particularly among patients who have had a myocardial infarction and arterial diseases, and it significantly affects self-esteem and overall psychological well-being. Despite significant advances in cardiac rehabilitation, the psychosocial aspects, especially those related to sexual health, remain underexplored. **Objectives:** The objective of this study was to examine the relationship between ED, self-esteem, and depression in male patients post-MI, and to assess how ED treatment affects patients’ psychological health. **Methods:** This cross-sectional study involved 80 male patients with a confirmed MI within the last six months, aged between 35 and 77 years. The Beck Depression Inventory-II (BDI-II) and Rosenberg Self-Esteem Scale (SES) were used to assess psychological well-being, while the International Index of Erectile Function-5 (IIEF-5) was employed specifically to evaluate ED. **Results:** This study demonstrates a strong interdependence between mental and physical health in post-myocardial infarction (MI) patients, with ED being a key factor affecting self-esteem. There is a significant negative correlation between self-esteem and depression, highlighting the importance of psychological resilience in post-MI rehabilitation. Elevated self-esteem helps mitigate depressive symptoms, contributing to improved mental health and rehabilitation outcomes. **Conclusions:** Older patients tend to have lower self-esteem, likely due to the presence of multiple comorbidities and a longer, more challenging recovery period post-MI. Age was a significant factor in predicting lower self-esteem, but other demographic characteristics did not have a strong influence on self-esteem, depression, or ED.

## 1. Introduction

Myocardial infarction (MI) represents a significant global health concern due to its high prevalence and substantial impact on patients’ lives. Recent systematic reviews and meta-analyses indicate that the global prevalence of MI varies significantly with age. In the population under 60 years old, the prevalence of MI is 3.8%, and this triples in patients aged >60. Moreover, it is estimated that approximately 9.5% of the general population aged over 60 years old has had a heart attack, highlighting the importance of this health problem, particularly in the context of aging societies [1]. Despite the overall decline in MI-related mortality rates in developed countries, there is a notable level of hospitalizations and long-term health consequences associated with heart failure, which often develop following an MI. In the United States alone, more than 1.1 million hospitalizations related to MI were reported in 2010, generating direct costs estimated to be approximately USD 450 billion. These data underscore the necessity for continued efforts to prevent and detect MI, particularly in high-risk populations [2,3].

Modern medicine increasingly acknowledges the complex relationship between physical and mental health, particularly in the context of chronic conditions like cardiovascular diseases (CVDs). Patients with heart conditions, whether congenital or acquired, often experience a diminished quality of life and face challenges in adapting to daily demands, which can significantly impact their self-esteem. Self-esteem, a central construct in psychology, is a critical resource that can shape patients’ perceptions of their illness and influence how they cope with its consequences [4]. CVD, as one of the leading causes of mortality worldwide, presents a significant challenge for modern medicine. Beyond the obvious physical consequences, heart disease can also lead to substantial psychological issues, including depression and reduced self-esteem. The literature increasingly emphasizes that psychological aspects of patient health, such as self-worth and mood, can directly influence the course of disease and the patient’s response to treatment [5]. Research indicates that individuals with severe forms of heart disease, particularly during adolescence, exhibit lower self-esteem and higher levels of depressive mood compared to patients with milder forms of the disease and healthy peers. This phenomenon suggests that self-esteem may be crucial in modulating cardiac patients’ subjective health experience and quality of life. The literature increasingly highlights that the subjective perception of disease severity and the accompanying negative emotions, rather than the physical symptoms alone, can significantly impact the psychological functioning of patients [6]. Low self-esteem may lead to an avoidance of social and sexual activities, further deteriorating the emotional state of patients. Moreover, erectile dysfunction (ED), often resulting from both physical and psychological factors associated with heart disease, can lead to a further decline in self-esteem, exacerbating depressive symptoms [4].

Depression is strongly associated with an increased risk of cardiovascular diseases, including MI, stroke, and heart failure. Research indicates that individuals with depression have a 28% higher risk of developing MI and a 16% higher risk of any cardiovascular disease. Moreover, depression significantly elevates the risk of cardiovascular and all-cause mortality, with the risk being exceptionally high in the case of heart failure, where it can increase up to threefold. Depression influences the development of CVD through both behavioral and physiological mechanisms. It can lead to unhealthy behaviors such as poor diet and physical inactivity, as well as to immunological disturbances, endothelial dysfunction, and dysregulation of the hypothalamic–pituitary–adrenal axis. These factors contribute to the development of atherosclerosis, hypertension, and other cardiovascular conditions. Early identification and treatment of depression can serve as effective preventive measures against cardiovascular disease [7,8].

The objective of this cross-sectional, partially prospective study was to explore the interconnections between depression, self-esteem, and ED in a population of male patients with a history of MI. Using validated tools such as Beck Depression Inventory-II (BDI-II) and Rosenberg Self-Esteem Scale (SES), this study aimed to provide a detailed analysis of whether psychological factors, such as self-esteem and depression, are correlated with sexual function in men after experiencing an MI.

## 2. Methodology

This study was a cross-sectional retrospective analysis that investigated the relationship between psychological factors, such as self-esteem and depression, and ED in post-MI patients. Patients who had experienced an MI were enrolled in the Cardiovascular Rehabilitation Outpatient Program (KOS). This program was designed to be a day rehabilitation unit, where patients would come from home to engage in two hours of supervised exercise sessions as part of their rehabilitation process. The rehabilitation program was non-residential, ensuring that patients remained in their home environment and returned daily for the prescribed exercise sessions. As part of the rehabilitation program, patients were asked to complete a series of psychological questionnaires. The forms were filled under the supervision of a trained psychologist to ensure accurate and reliable responses. The collected data were used to assess the psychological well-being of the patients, including measures of depression, anxiety, and perceived stress. The questionnaires were an integral part of the rehabilitation program, helping to tailor the therapeutic approach to each patient’s needs. The study was conducted in accordance with the Declaration of Helsinki and approved by the Ethics Committee of Pomeranian Medical University KB-00/10/2022 in October 2022.

The study included patients admitted to the rehabilitation facility as part of a recovery program after being treated for an MI within the past six months. The study group included patients aged between 35 and 77 years at the time of MI. Eighty male patients were provided with the questionnaires. Two patients who did not return all questionnaires were excluded from the study. Patients who reported a lack of sexual activity for six months prior to their MI due to decreased libido or other unspecified reasons were excluded from the study. This criterion aimed to eliminate patients whose reduced sexual function might have been caused by factors unrelated to ED, which could otherwise confound the interpretation of the relationship between ED and psychological variables. Several questionnaires were used to assess different psychological outcomes and measures, and they are described below.

The Beck Depression Inventory (BDI) is a widely used self-report tool designed to assess the severity of depression in adults. Initially developed in 1961, the BDI consists of 21 items, each rated on a scale from 0 to 3, producing a total score ranging from 0 to 63. The BDI was designed as a quantitative measure of depression rather than a diagnostic tool. The BDI’s psychometric properties are robust, with high internal consistency (Cronbach’s alpha ranging from 0.82 to 0.91) and good test–retest reliability. Various versions, including the BDI-II and BDI-Fast Screen, address specific populations, such as older adults and medical patients. The BDI remains a valuable tool in both clinical and research settings for screening and monitoring depression, particularly in adult and geriatric populations. However, its use in cognitively impaired individuals may lead to higher false-positive rates, and somatic symptoms can complicate score interpretation. In this study, the BDI was specifically chosen for its ability to quantitatively assess depressive symptoms in a population that may exhibit both psychological and somatic responses to their condition, allowing for a comprehensive understanding of depression’s impact on patient outcomes [9].

The Rosenberg Self-Esteem Scale (SES) was included in this study to measure self-esteem, a critical psychological factor influencing recovery and quality of life in patients with chronic conditions. The SES, initially developed by Morris Rosenberg in 1965, consists of ten statements rated on a 4-point Likert scale, ranging from “strongly agree” to “strongly disagree”. This scale assesses global self-worth by measuring positive and negative feelings about the self. Scores range from 10 to 40, with higher scores indicating higher self-esteem. The SES has demonstrated good reliability, with Cronbach’s alpha values typically ranging from 0.77 to 0.88. It has been validated in numerous studies across various populations, ensuring that it provides reliable and meaningful data when assessing self-esteem in individuals recovering from serious cardiovascular events. In this study, the SES scale was selected to evaluate self-esteem’s impact on patients’ psychological well-being after an MI [10].

The International Index of Erectile Function (IIEF-5) questionnaire was included to assess the presence and severity of ED in men, with ED being a common issue that can significantly affect quality of life following MI. The IIEF-5 is a shortened form of the original 15-item IIEF, consisting of five questions focusing on critical areas of erectile function over the four weeks prior to completion of the questionnaire. Respondents rate their experiences using a Likert scale, where higher scores indicate better erectile function. The IIEF-5 scores range from 5 to 25, with lower scores indicating more severe ED. The IIEF-5 has demonstrated high reliability, with Cronbach’s alpha values typically ranging from 0.88 to 0.92. It is widely used in clinical settings and research due to its reliability and ease of use. In this study, the IIEF-5 was selected due to its specificity and sensitivity in detecting ED in a clinical setting, particularly in men with a history of MI. This tool was critical for exploring the relationship between cardiovascular health and sexual function, providing insights that could guide future interventions to improve psychological and physical health outcomes [11].

### 2.1. Study Demographics

Eighty patients were enrolled in the study, ranging in age from 35 to 77 years (M = 57.06; SD = 10.12). Patient demographics are summarized below in Table 1.

### 2.2. Statistical Analysis

To verify the research hypotheses, statistical analyses were conducted using IBM SPSS version 25. Descriptive statistics, Shapiro–Wilk tests, Pearson’s correlation, Kruskal–Wallis tests, Mann–Whitney U tests, and Spearman’s rank correlation were performed. A significance level of α = 0.05 was applied.

## 3. Results

Adhering to the statistical assumptions, a full set of descriptive statistics was calculated for the quantitative variables, alongside Shapiro–Wilk tests for normality. The age distribution of the patients approximated a Gaussian distribution. For other variables, non-normal distributions were observed, as indicated by significant Shapiro–Wilk test results (see Table 2). However, skewness values for all variables fell within the range of −2 to +2, suggesting no significant asymmetry. Consequently, parametric tests were used, provided their other assumptions were met, ensuring the validity of the results.

### 3.1. Clinical Characteristics

Within the study group, 73.8% of patients (59 individuals) had chronic cardiac conditions, including hypertension (60 patients), dyspnea or chest pain (19 patients), and cardiac arrhythmia (14 patients). As part of their medical treatment, five patients used nitroglycerin, and twelve (15%) used isosorbide mononitrate. Only one patient reported using any form of pharmacotherapy for ED.

### 3.2. Self-Esteem in Post-MI Patients

**Figure 1:**
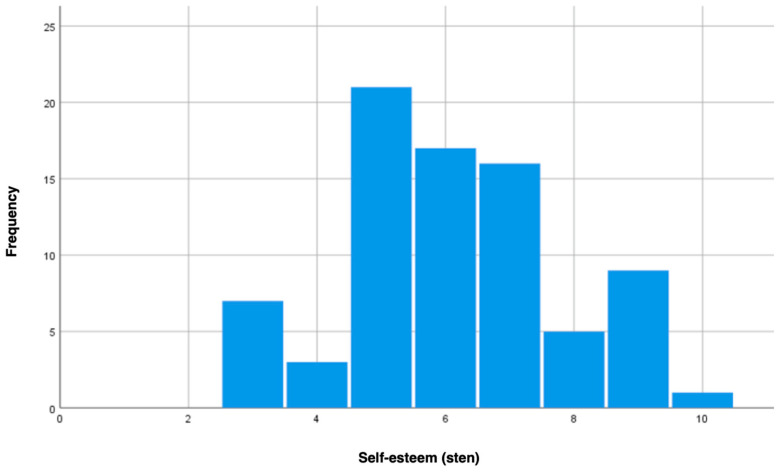
Self-esteem of post-MI patients.

As part of this study, we assessed the self-esteem levels of patients treated at the rehabilitation facility after an MI. Raw SES questionnaire scores were converted to standard scores using Polish norms; the results are illustrated in Figure 1. Most of the questioned patients scored within the average range (sten scores of 5–6), with a noticeable skew towards higher scores (sten scores of 8–10) compared to lower scores (sten scores of 1–3). No participant scored in the lowest range (sten scores of 1–2), which may suggest generally average to high self-esteem levels among the examined patients.

### 3.3. Erectile Function in Post-MI Patients

**Figure 2:**
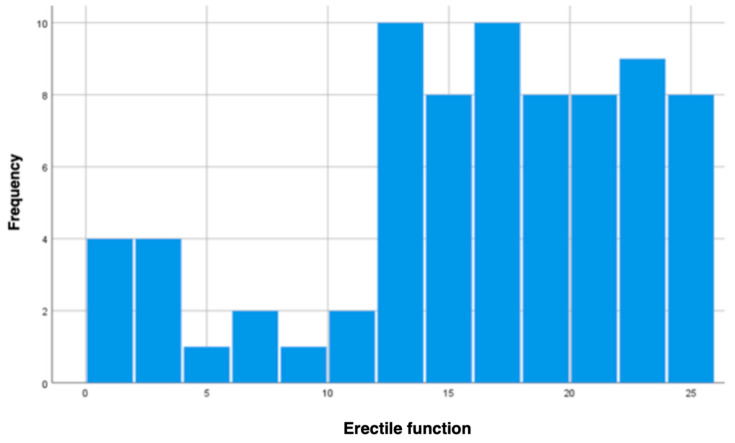
Erectile function assessment.

In this study, we also examined patients’ erectile function using the IIEF-5. Patients scored from 0 to 25 points. As demonstrated in Figure 2, most of the assessed patients scored within the average to high range.

### 3.4. Depression Levels in Post-MI Patients

**Figure 3:**
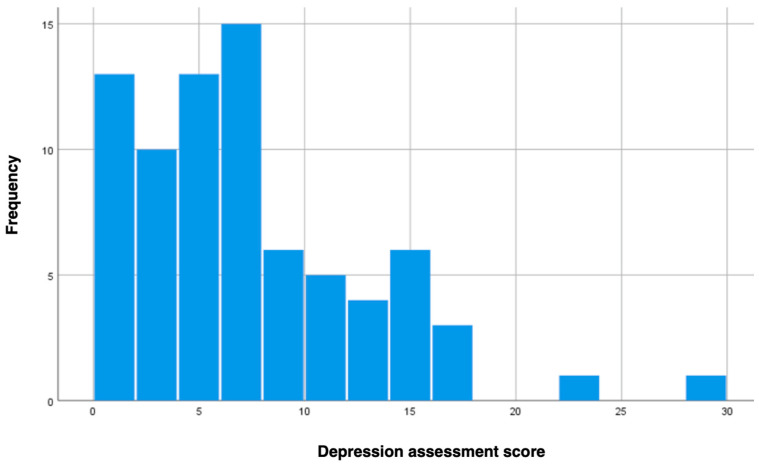
Depression assessment among post-MI patients.

The depression levels were assessed using the following scale: patients who scored 0–11 points were assessed as having no depression/dysthymia, 12–19 as having mild depression, 20–25 as having moderate depression, and scores over 26 as having severe depression. Most participants were classified as having no depression or dysthymia (80.5% of the sample). Only a few individuals showed signs of moderate or severe depression, as depicted in Figure 3.

### 3.5. Correlation between Self-Esteem and Erectile Functioning

**Figure 4:**
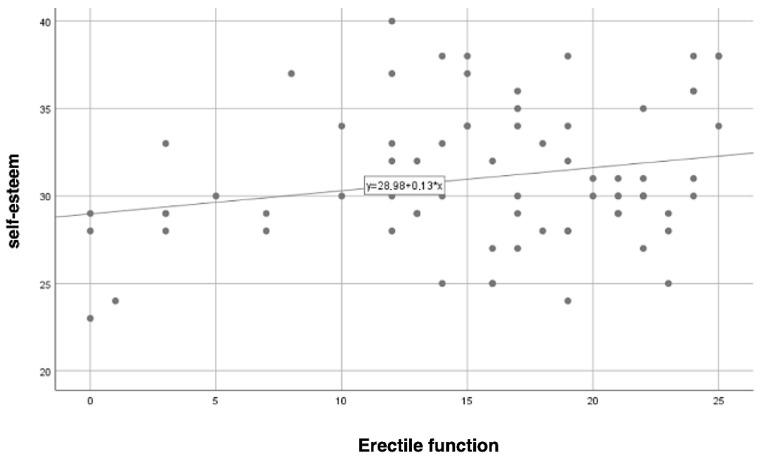
The correlation between erectile function and patients’ self-esteem.

Pearson’s correlation analysis revealed a statistically significant relationship between self-esteem and ED (r = 0.23; *p* = 0.050). As illustrated in Figure 4, higher self-esteem was associated with fewer instances of ED, although the strength of this relationship was low.

### 3.6. Correlation between Self-Esteem and Depression Levels

**Figure 5:**
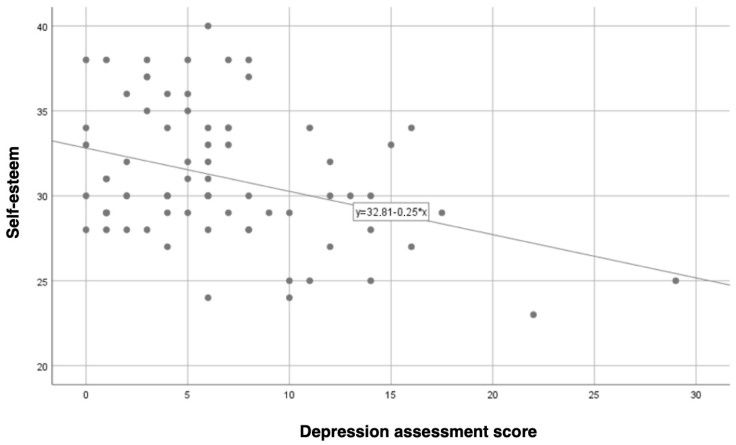
The correlation between depression assessment score and patients’ self-esteem.

Further analysis demonstrated a statistically significant negative correlation between self-esteem and depression levels (r = −0.36; *p* = 0.001). As shown in Figure 5, higher self-esteem was associated with lower depression levels, with the strength of this relationship being moderately strong.

### 3.7. Other Demographic Variables

This study also explored the relationships between various demographic factors and self-esteem, depression, and sexual activity quality. Age was negatively correlated with self-esteem (r = −0.25; *p* < 0.05), indicating that older patients tended to have lower self-esteem. However, no significant correlations were found between self-esteem, depression, sexual activity quality, and other variables like the size of the place of residence, educational level, marital status, or occupational status.

## 4. Discussion

Depression and reduced self-esteem are common consequences of MI and negatively affect both patient recovery and general prognosis, including through heightened mortality [12]. Rehabilitation programs for post-MI patients were created in order to quicken patients’ recovery and allow self-functioning. Not only the physical changes that occur to one’s functioning impact patients’ prognosis but also their mental health. Following an unexpected MI, patients face various challenges, including health-related fears, lifestyle changes, financial stress, and social adjustments.

ED is a common male sexual problem that significantly affects quality of life, not only of the men but also of their families. It is estimated that approximately 150 million men are affected by ED, and this number is estimated to double by 2025 [13]. ED, once supposed to be of psychological etiology, is now considered to be a dysfunction of vascular origin, often associated with patients’ chronic cardiovascular comorbidities. Increasing atherosclerosis caused by CVD subsequently leads to the disruption of endothelial integrity and impaired vasodilation, causing problems in erectile processes [14,15].

The results of our study provide strong evidence for the interdependence between the mental and physical health of patients after MI. Our analyses revealed a significant decrease in self-esteem among patients who have ED, which supported the initial study hypothesis. As the presence of ED is common among patients after MI, it may be another risk factor for lower self-esteem in patients during the period of cardiac rehabilitation [16,17,18].

Our study highlights a significant relationship between higher self-esteem and lower levels of depression in post-MI patients. This finding is crucial in the context of cardiac rehabilitation, emphasizing the need for a comprehensive approach that addresses not only physical recovery but also psychological resilience. Elevated self-esteem may play a pivotal role in mitigating depressive symptoms, which in turn contributes to improved overall mental health and coping mechanisms during rehabilitation. Incorporating strategies to enhance self-esteem within rehabilitation programs could therefore significantly improve quality of life for patients recovering from an MI [19,20,21].

Furthermore, our study confirmed that the presence of chronic CVD has a significant impact on the frequency of ED. Patients with a medical history of chronic CVD before the onset of MI were observed to have suffered from ED more frequently when compared to the group of patients without any cardiovascular comorbidities.

The results of our study align with the triad model of depressive disorders, cardiovascular symptoms, and ED, which postulates mutually reinforcing connections between these conditions. The literature frequently highlights that depression, cardiovascular disease, and ED share common risk factors, such as hypertension, obesity, and smoking, indicating a strong link between them. According to this model, patients presenting with ED symptoms should be routinely evaluated for depression and cardiovascular disease, and those with depression should be assessed for ED and cardiac risk. In the context of our findings, which demonstrated a significant association between lower self-esteem and the presence of ED, it is essential to emphasize the need for a comprehensive approach to the care of post-MI patients. Addressing both physical and psychological aspects could significantly improve quality of life and reduce the risk of further cardiovascular complications and depression in this patient group [22,23]. The findings of this study are consistent with the conclusions of Corona et al. (2020), who propose that ED is not only a consequence of CVD but also an early indicator of forthcoming cardiovascular events. Moreover, just as Corona et al. identified a significant relationship between ED and depressive symptoms, our study highlights the psychological burden of ED, particularly its association with lower self-esteem and higher depression scores. These findings support the necessity of including sexual health assessments as a routine part of cardiac rehabilitation, in line with the proposed comprehensive care model for reducing cardiovascular risk [24]. Our study aligns with Ziapour et al. (2024), which reported the global prevalence of sexual dysfunction in cardiovascular patients to be 62.6%. Both studies emphasize the strong connection between cardiovascular disease and sexual dysfunction, particularly ED. Our findings of significant links between ED, depression, and low self-esteem echo Ziapour et al.’s observation that mental health plays a key role in sexual dysfunction among heart patients. These results reinforce the need for a comprehensive approach to cardiac rehabilitation that includes both physical and psychological care to improve overall recovery and quality of life [25].

The findings of our study have shown the influence of patients’ age on self-esteem, with older patients tending to have lower self-esteem. This may be due to the presence of multiple comorbidities and a more difficult, elongated period of time required for full recovery after an MI and for returning to their previous medical state. Advanced patient age is likely to be associated with unfavorable post-MI cardiac structural changes and higher mortality rates. As older patients tend to have more comorbidities, the treatment of other chronic cardiovascular conditions such as hypertension, diabetes, and hyperlipidemia is also more difficult to control, especially if these conditions were untreated before the MI [26].

Patients’ demographic characteristics, other than patients’ age, did not seem to influence patients’ self-esteem, depression, or ED scores.

### Limitations

This study has several notable limitations. First, this study did not assess testosterone levels among participants, which are known to significantly impact erectile function. This omission may limit the understanding of sexual dysfunction within the analyzed group. Future research should include testosterone measurements to provide a more comprehensive picture of sexual health. Second, this study was conducted within a single rehabilitation unit on a relatively small and homogeneous patient group, which may limit the generalizability of the findings to a broader population. Expanding the study to a larger scale would enhance its validity. Third, measurements were taken only once, which may not capture long-term changes in the patients’ mental and physical health. Additionally, the study did not account for the long-term effects of treatments, such as ED therapy, on patient outcomes.

## 5. Conclusions

Among post-MI patients, the presence of ED was significantly associated with lower self-esteem and higher levels of depression. These findings emphasize the importance of an integrated rehabilitation approach that addresses both mental health and sexual health. While these results offer valuable insights into the interaction between physical and mental health, further research is needed to evaluate the clinical effectiveness of such interventions and better understand their impact on patients’ psychological resilience and quality of life.

## Figures and Tables

**Table 1:** Demographic characteristics of study participants.

Variable	N	%
Place of residence		
Village	13	16.3%
Town < 50,000 inhabitants	11	13.8%
Town 50,000–150,000 inhabitants	3	3.8%
City 150,000–500,000 inhabitants	48	60%
No data	5	6.3%
Educational level		
Primary	1	1.3%
Secondary	23	28.7%
Technical/vocational	32	40%
Higher education	23	28.7%
No data	1	1.3%
Marital status		
Married	57	71.3%
Unmarried partnership	9	11.3%
Widowed	2	2.5%
Single	11	13.8%
No data	1	1.3%
Occupational status		
Retired	23	28.7%
Employed	51	63.7%
No data	6	7.5%

**Table 2:** Descriptive statistics for the quantitative variables.

Variable	M	Me	SD	Sk.	Kurt.	Min	Max	W	*p*
Age	57.06	57.50	10.12	−0.19	−0.80	35	77	0.98	0.202
Self-Esteem (SES)	31.05	30	3.87	0.31	−0.45	23	40	0.96	0.019
Erectile Function (IIEF-5)	15.71	17	6.79	−0.78	−0.12	0	25	0.93	<0.001
Depression Level (BDI-II)	6.80	6	5.54	1.34	2.54	0	29	0.90	<0.001

## Data Availability

Data are available upon request from the first author.

## References

[1] Salari N., Morddarvanjoghi F., Abdolmaleki A., Rasoulpoor S., Khaleghi A.A., Hezarkhani L.A., Shohaimi S., Mohammadi M. (2023). The global prevalence of myocardial infarction: A systematic review and meta-analysis. BMC Cardiovasc. Disord..

[2] Gerber Y., Weston S.A., Jiang R., Roger V.L. (2014). The changing epidemiology of myocardial infarction in olmsted county, minnesota, 1995–2012. Am. J. Med..

[3] Shang H., Chen J., Zhang J., Xiang Y., Cao H., Ren M., Wang H., Xu H., Li J., Liu D. (2009). Three therapeutic tendencies for secondary prevention of myocardial infarction and possible role of Chinese traditional patent medicine: Viewpoint of evidence-based medicine. J. Evid.-Based Med..

[4] Qin Z., Mei S., Gao T., Liang L., Li C., Hu Y., Guo X., Meng C., Lv J., Yuan T. (2021). Self-esteem as a mediator between life satisfaction and depression among cardiovascular disease patients. Clin. Nurs. Res..

[5] Fan R.R., Rudnick S.B., Minami H.R., Chen A.M., Zemela M.S., Wittgen C.M., Williams M.S., Smeds M.R. (2022). Depression screening in patients with vascular disease. Vascular.

[6] Cohen M., Mansoor D., Langut H., Lorber A. (2007). Quality of Life, Depressed Mood, and Self-Esteem in Adolescents With Heart Disease. Psychosom. Med..

[7] Lin Y.-H., Lin Y.-H. (2022). Mental stress–induced myocardial ischemia and cardiovascular events in patients with coronary heart disease. JAMA.

[8] Harshfield E.L., Pennells L., Schwartz J.E., Willeit P., Kaptoge S., Bell S., Shaffer J.A., Bolton T., Spackman S., Wassertheil-Smoller S. (2020). Association between depressive symptoms and incident cardiovascular diseases. JAMA.

[9] Vaccarino V., Almuwaqqat Z., Kim J.H., Hammadah M., Shah A.J., Ko Y.-A., Elon L., Sullivan S., Shah A., Alkhoder A. (2021). Association of mental stress–induced myocardial ischemia with cardiovascular events in patients with coronary heart disease. JAMA.

[10] Dzwonkowska I., Lachowicz-Tabaczek K., Łaguna M. (2007). Skala samooceny SES Morrisa Rosenberga–polska adaptacja metody. Psychol. Społeczna.

[11] Rosen R., Cappelleri J., Smith M.D., Lipsky J., Peña B. (1999). Development and evaluation of an abridged, 5-item version of the International Index of Erectile Function (IIEF-5) as a diagnostic tool for erectile dysfunction. Int. J. Impot. Res..

[12] Feng L., Li L., Liu W., Yang J., Wang Q., Shi L., Luo M. (2019). Prevalence of depression in myocardial infarction: A PRISMA-compliant meta-analysis. Medicine.

[13] Aytaç, McKinlay J.B., Krane R.J. (1999). The likely worldwide increase in erectile dysfunction between 1995 and 2025 and some possible policy consequences. BJU Int..

[14] Montorsi P., Montorsi F., Schulman C.C. (2003). Is erectile dysfunction the “tip of the iceberg” of a systemic vascular disorder?. Eur. Urol..

[15] World Health Organization (2007). Prevention of Cardiovascular Disease: Guidelines for Assessment and Management of Total Cardiovascular Risk.

[16] Hooshang H., Farahani A.V., Rezaeizadeh H., Forouzannia S.K., Alaeddini F., Ashraf H., Karimi M. (2022). Efficacy of date palm pollen in the male sexual dysfunction after coronary artery bypass graft: A randomized, double-blind, clinical trial. Evid.-Based Complement. Altern. Med..

[17] Tor-Anyiin I., Omokhua O.E., Swende L.T. (2024). Association between erectile dysfunction and cardiovascular risk factors in a nigeria tertiary hospital. Rwanda Med. J..

[18] Li J.Z., Maguire T.A., Zou K.H., Lee L.J., Donde S.S., Taylor D.G. (2022). Prevalence, comorbidities, and risk factors of erectile dysfunction: Results from a prospective real-world study in the United Kingdom. Int. J. Clin. Pract..

[19] El-Osta A., Kerr G., Alaa A., El Asmar M.L., Karki M., Webber I., Sasco E.R., Blume G., Beecken W.-D., Mummery D. (2023). Investigating self-reported efficacy of lifestyle medicine approaches to tackle erectile dysfunction: A cross-sectional esurvey based study. BMC Urol..

[20] Katz A. (2021). Erectile dysfunction treatment improves self-esteem and quality of life in men with cardiovascular disease. J. Sex. Med..

[21] Shteynshlyuger A., Kaufman M. (2021). Comparative effectiveness of different treatments for erectile dysfunction: Implications for improving self-esteem in post-MI patients. Int. J. Impot. Res..

[22] Goldstein I. (2000). The mutually reinforcing triad of depressive symptoms, cardiovascular disease, and erectile dysfunction. Am. J. Cardiol..

[23] Amadio P., Zarà M., Sandrini L., Ieraci A., Barbieri S.S. (2020). Depression and Cardiovascular Disease: The Viewpoint of Platelets. Int. J. Mol. Sci..

[24] Corona G., Rastrelli G., Isidori A., Pivonello R., Bettocchi C., Reisman Y., Sforza A., Maggi M. (2020). Erectile dysfunction and cardiovascular risk: A review of current findings. Expert Rev. Cardiovasc. Ther..

[25] Ziapour A., Kazeminia M., Rouzbahani M., Bakhshi S., Montazeri N., Yıldırım M., Tadbiri H., Moradi F., Janjani P. (2024). Global prevalence of sexual dysfunction in cardiovascular patients: A systematic review and meta-analysis. Syst. Rev..

[26] Shih J.-Y., Chen Z.-C., Chang H.-Y., Liu Y.-W., Ho C.-H., Chang W.-T. (2019). Risks of age and sex on clinical outcomes post myocardial infarction. IJC Hear. Vasc..

